# Hyper-Dense_Lung_Seg: Multimodal-Fusion-Based Modified U-Net for Lung Tumour Segmentation Using Multimodality of CT-PET Scans

**DOI:** 10.3390/diagnostics13223481

**Published:** 2023-11-20

**Authors:** Goram Mufarah Alshmrani, Qiang Ni, Richard Jiang, Nada Muhammed

**Affiliations:** 1 School of Computing and Commutations, Lancaster University, Lancaster LA1 4YW, UK; q.ni@lancaster.ac.uk (Q.N.); r.jiang2@lancaster.ac.uk (R.J.); 2 College of Computing and Information Technology, University of Bisha, Bisha 67714, Saudi Arabia; 3 Computers and Control Engineering Department, Faculty of Engineering, Tanta University, Tanta 31733, Egypt; nada_elshennawy@f-eng.tanta.edu.eg

**Keywords:** AI, U-Net, multimodal fusion, hyper-dense, multimodality imaging CT, PET, lung tumour segmentation

## Abstract

The majority of cancer-related deaths globally are due to lung cancer, which also has the second-highest mortality rate. The segmentation of lung tumours, treatment evaluation, and tumour stage classification have become significantly more accessible with the advent of PET/CT scans. With the advent of PET/CT scans, it is possible to obtain both functioning and anatomic data during a single examination. However, integrating images from different modalities can indeed be time-consuming for medical professionals and remains a challenging task. This challenge arises from several factors, including differences in image acquisition techniques, image resolutions, and the inherent variations in the spectral and temporal data captured by different imaging modalities. Artificial Intelligence (AI) methodologies have shown potential in the automation of image integration and segmentation. To address these challenges, multimodal fusion approach-based U-Net architecture (early fusion, late fusion, dense fusion, hyper-dense fusion, and hyper-dense VGG16 U-Net) are proposed for lung tumour segmentation. Dice scores of 73% show that hyper-dense VGG16 U-Net is superior to the other four proposed models. The proposed method can potentially aid medical professionals in detecting lung cancer at an early stage.

## 1. Introduction

In 2020, lung cancer ranked as the second most prevalent cancer, accounting for around 11.4% of all newly identified instances of cancer, with approximately 2.2 million individuals affected. Furthermore, it was the primary cause of cancer-related mortality, responsible for approximately 18.0% of the deaths caused by cancer globally, resulting in approximately 1.8 million fatalities. Loss of appetite, exhaustion, chronic coughing, and chest pain are among the symptoms of lung cancer, which can cause unimaginable anguish for the sufferer [1].

The segmentation of lung tumours, treatment evaluation, and tumour stage classification have become significantly more accessible with the advent of PET/CT scans. Moreover, the molecular characteristics and anatomic aberrations of the target lesion can be observed with PET/CT. The PET imaging technique does not involve cutting or surgery. By detecting illness markers earlier, PET allows for earlier diagnosis than imaging modalities like MRI and CT [2]. Their metabolic processes can be analysed for their physiological function and biochemical features by studying particular organs and tissues. PET can detect molecular and cellular levels of tissue metabolism.

However, multimodality imaging technology, such as PET-CT scanners, has simultaneously made it possible to record functional and anatomical information [3]. It is a rigorous and time-consuming process for oncologists, radiologists, and pulmonologists to manually segment the lesions and tumours, leading to delays in therapy and decreased survival rates, particularly in clinics with insufficient resources. In addition, specialist knowledge and clinical experience are necessary for high-quality manual localization and segmentation. Because of this, computer-aided diagnostic (CAD) systems [4] were developed to replace radiologists’ manual viewing of lung cancer. Combining lung segmentation approaches with radiologists’ knowledge can reduce the burden on radiologists and boost their productivity and accuracy. Many recent advancements in image segmentation have allowed for more precise and effective treatment and diagnosis. Thresholding, Atlas, and Region Growing are some examples of classic automatic segmentation methods. These approaches use the shallow qualities of an image, such as grayscale, texture, gradient, and many more, to segment the object [5]. However, conventional segmentation techniques have difficulty distinguishing between tumours and surrounding healthy tissue because their intensity distributions are similar. In addition, these tasks typically involve manual processes and are characterized by a significant investment of time. Moreover, they are subject to substantial heterogeneity across operators.

Furthermore, the complexity of the background in CT images consistently provides quite different information when comparing PET and CT scans. As a result of these constraints, deep-learning-based algorithms have proven to be superior in auto-segmenting medical images [6].

Deep learning (DL) models automatically extract features and apply the learned high-dimensional abstractions for performing segmentation. The effectiveness of fully convolutional networks (FCN) for semantic segmentation is promising [7]. In an FCN, the fully connected layer is replaced by a convolutional layer. This comprehensive framework has served as the foundation for subsequent studies of semantic segmentation of medical images. Medical image segmentation commonly uses U-Net [8], built on the FCN architecture. Using skip-connection architecture, each layer’s down-sampled features are joined with their up-sampled counterparts. This mechanism is similar to an encoder-decoder, but it is more effective and does not require a great deal of disk space. FCN-based networks, such as U-Net, have surpassed manual or semi-automatic segmentation methods since the emergence of big data methods.

The U-Net architecture is a convolutional neural network (CNN) primarily used to recognize image patterns. U-Net semantic segmentation relies extensively on the categorization of image pixels. Segmenting lung tumours can be reduced to a foreground/background pixel binary classification problem. The down-sampling and up-sampling module is responsible for the U-Net architecture. The surface layer is where localization information is learned, but the down-sampling procedure, also known as the pooling procedure, may improve the volume of context data the network learns [8].

The VGG model investigates the effect of convolutional network depth on recognition accuracy in a large-scale setting. Our main contribution is a thorough evaluation of increasing-depth networks using an architecture with 3 × 3 convolution filters, which shows a significant improvement. The VGG model, a kind of CNN, was created to improve model performance when more layers are added. The VGG model takes 224 × 224 colour images as its primary input and feeds them via a sequence of convolutional layers, with filter sizes of 3 × 3 and 1 × 1 with the stride of 1 and valid padding, as well as max pooling with 2 × 2 with the stride of 2. Finally, we have a three-layer network with a SoftMax activation function and 4096 neurons in the first two layers, followed by 1000 neurons in the last layer. VGG [9] presents the two primary models, VGG16 and VGG19.

In comparison to the VGG-19 network, which has 19 layers of typical convolutional networks, the VGG-16 network [9] only has 16, with filter sizes of 3 × 3 and strides of 1. Each of the five blocks is separated from the next by a max-pooling layer. There are three interconnected layers on top of the blocks.

The significant contributions of this research are given below.

- The inputs to the proposed architecture are PET and CT scans. Here, dense connections happen along the same pathways that process each modality individually. Finally, their features are joined together at a high layer to finish separating them.
- Five deep models based on U-Net architecture are suggested for lung cancer segmentation in multimodal image scenarios: early fusion, late fusion, dense fusion, hyper-dense fusion, and VGG-16 U-Net.
- The performance of the suggested models was evaluated using three types of loss functions: binary, Dice, and focal loss functions.

The remainder of the paper is structured as follows. Section 2 outlines studies on multimodal PET-CT images for segmentation based on deep learning. The proposed network architecture is described in Section 3. In Section 4, the results of the experiments and their analysis are presented. Section 5 presents the study’s findings and recommendations for moving forward.

## 2. Literature Review

CT and PET imaging are used in various research papers because of the unique insights they provide into the structure and function of the human body, respectively. Combining the two allows for the early detection of even the tiniest lung tumours.

Wang et al. [10] advised a DL-based dual-modality approach using CT and PET scans to develop an automated segmenting of lung tumours for radiation therapy planning. Two distinct convolution routes were built into the 3D convolutional neural network for extracting features at different resolutions from the PETs and simulated CTs, and a single deconvolution path was also built into the network. Tumour segmentation via skip connections at each granularity was achieved by aggregating the obtained characteristics from the convolution arms and feeding them into the deconvolution pathway. A panel of oncologists judged the medical effectiveness of the network-generated segmentation strategy. While this work has many promising applications, it does have some caveats. The network may struggle to produce precise segmentations when tumour edges are not precise on CT or PET.

Park et al. [11] presented a two-stage U-Net model to boost the segmentation effectiveness of lung tumours by utilizing [18F]FDG PET/CT, as precise segmentation is necessary for determining the functional size of a tumour in this imaging modality. The LifeX program was used to create the tumour volume of interest. In the first step, a 3D PET/CT volume is used to train a global U-Net, based on which a 3D binary volume is then retrieved to serve as an initial representation of the tumour’s region. In the second stage, the PET/CT slice identified in Stage 1 is sent to the U-Net, generating a 2D binary image centred on the eight adjacent slices. The major drawback of the research is the lack of a 3D volume as the final result of the suggested approach. It may cause the coronal and sagittal slices to have gaps between the binary segments.

Xiang et al. [12] recommended a modality-specific segmentation network (MoSNet) to segment lung tumours. To better understand the differences between PET and CT scans, MoSNet is taught to use modality-specific representations, while modality-fused representations are employed to convert the typical characteristics of lung tumours in both scan types. The authors suggest an adversarial approach that uses an adversarial purpose concerning a modality discriminator and a reserved modality-common illustration to reduce the modality difference’s approximation. As a result, the network’s ability to represent data for the segmentation in PET and CT scans is enhanced. By generating a map for each modality, MoSNet can explicitly quantify the weights for the attributes in each modality. However, the limitation of the research is that the proposed approach is developed for 2D thorax PET-CT slices.

Fu et al. [13] proposed a DL system for lung cancer segmentation, i.e., a multimodal spatial attention module (MSAM). It is trained to highlight tumour-related regions selectively and downplay those physiologically rising from the PET scans. Next, using the created spatial attention maps, a CNN core is trained to focus on areas of a CT image with a higher propensity for tumours. The drawback of the research is that the datasets used only had one observer defining the outlines. If numerous observers had been used to reach a consensus segmentation, things would have gone much smoother. Because of the potential vagueness of the related thresholding approach used to create the ground truth for the NSCLC dataset, the segmentation outputs require human adjustment to correct for incorrectly categorized ROIs.

Zhong et al. [14] provided an innovative method for lung tumour segmentation by bringing together a robust FCN-based 3D U-Net and a graph-cut-based co-segmentation model. Initially, high-level discriminative features for PET and CT images are learned by independently training two distinct deep U-Nets on the datasets. These features then create tumour/non-tumour masks and probability maps. The final tumour segmentation findings are obtained using the PET and CT probability mappings in a graph-cut-based co-segmentation model. Despite fusing their extracted features, the research has given different results for CT and PET.

Hwang et al. [15] recommended a new network architecture called 3C-Net, which uses numerous contexts in three distinct ways. Two decoders in the network are implemented to exploit inter-slice contextual information: a segmentation decoder and a context decoder. The context decoder receives the inter-slice difference features and uses them to predict the segmentation mask’s inter-slice difference. Having this 3D background information for each slice helps in attention direction. The prediction results from each decoder stage are used to derive a loss function for network optimization. Since two modalities are used, i.e., PET/CT data, a co-encoder block is implemented to extract mutually reinforcing features from both modalities while simultaneously acquiring contextual knowledge about them. Weights for both CT and PET are modified twice in co-encoder blocks. The co-encoder blocks take in relevant data from both modalities, allowing for interaction while maintaining spatial and structural coherence. The encoder additionally includes an asterisk spatial pyramid pooling (ASPP) block in its final step. The ASPP block aids the network in increasing the scope of its observations and avoiding the loss of spatial context, which allows the recording of visual context at various scales.

Kumar et al. [16] improved the multimodality PET-CT fusion using CNN, which learns to fuse complementary information. The proposed CNN stores modality-specific characteristics before deriving a spatially variable fusion map. It allows quantifying the relevance of each modality’s characteristics across various spots. Moreover, multiplying the fusion maps with the modality-specific feature maps yields representations of the complementary multimodality data at various positions. The recommended CNN is tested on PET-CT scans of lung tumours, where its ability to detect and separate many regions with variable fusion needs is evaluated.

Jemaa et al. [17] demonstrated a comprehensive strategy employing 2D and 3D CNN for rapid tumour classification and metabolic data retrieval from whole-body FDG-PET/CT images. This architecture is relatively economical between tumour load and healthy tissue volume, and between the intrinsic heterogeneity of the input images, which is especially important for whole-body scans due to their vast size and high asymmetry.

Zhao et al. [18] developed a novel multimodality segmentation approach that utilizes a 3D FCN, and simultaneously includes PET and CT data in tumour segmentation. Initially, the network underwent a multitask training phase, during which two parallel sub-segmentation architectures, each built with a deep CNN, were learned to generate map-like features from both modalities. The PET/CT feature maps’ characteristics were re-extracted using a weighted cross-entropy reduction technique, and a feature fusion component was then constructed using cascaded convolutional modules. The SoftMax function was also used to generate the cancer mask as the network’s final output. The research lacks an automatic setting of the weighting parameters of the loss functions, which can affect performance. Also, more effective ways for feature extraction can increase the performance of the segmentation.

Using W-net, Zhong et al. [19] evaluated 3D deep fully convolutional networks (DFCN) for tumour co-segmentation on dual-modality NSCLC and PET-CT images. CT and PET data are combined to better understand NSCLC tumours in PET-CT scans and apply DFCN co-segmentation. The recommended DFCN-based co-segmentation approach uses two connected 3D U-Nets with an encoder-decoder to exchange complementing data between PET and CT.

Bi et al. [20] developed a hyper-connected fusion model that uses a CNN-TN fusion encoder and a CNN-TN fusion decoder. With hyper connections between them, the encoder splits into three forks to independently handle PET, CT, and combined PET-CT scans. The transformer encoders process the encoded image embeddings to learn complimentary characteristics in a long-range dependency between the PET, CT, and concatenated PET-CT images. The transformer decoder combines the learnt embeddings to find characteristics important for segmentation, which are subsequently transformed into a 2D feature map. The segmentation results are then up-sampled using a convolutional neural network. The data came from the soft-tissue sarcoma databases. The data showed that the model’s Dice had a probability of 66.36%. The summaries of the literature research in lung tumour segmentation models are listed in Table 1.

## 3. Methodology

This section discusses the segmentation models for lung cancer in depth. The underlying functions of the proposed models are also described. The U-Net technique is the basis for the proposed models; it has demonstrated usefulness in medical image segmentation. A U-Net model is fed the CT and PET scan data. During the decoder step, further improvement is performed in the model’s recognition of the input image features by creating dense connections across layers in the same input branch of the U-Net.

**Figure 1:**
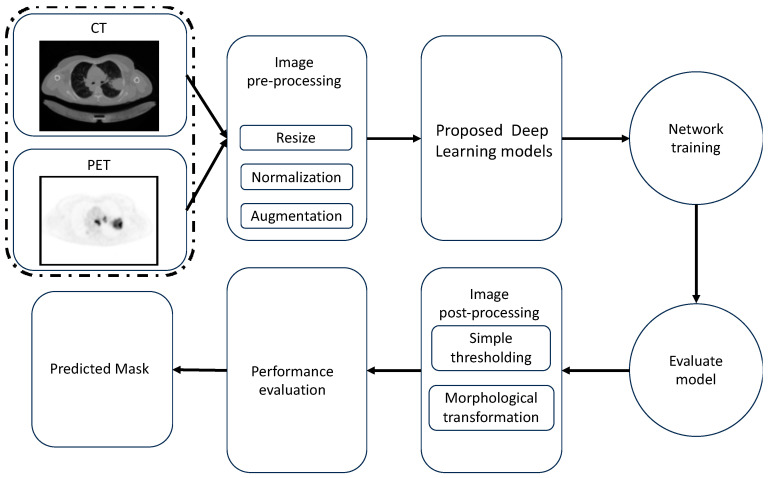
Block diagram for the lung cancer segmentation framework.

The proposed architecture for lung cancer segmentation is shown in Figure 1. Figure 1 depicts the three main stages of our framework: (1) image pre-processing, (2) multimodality U-Net segmentation, and (3) medical image post-processing. The pre-processing, augmentation, and post-processing methods are discussed in the following subsections.

### 3.1. Image Processing

#### 3.1.1. Image Pre-Processing

The intensity levels of the image’s pixels were normalized. In addition to resizing each image, the pixel scale value was changed from (0–255) to (0–1) to reduce the level of complexity of the images. To simplify model training, we decreased the resolution of the CT and PET scans in the dataset to 256 × 256 pixels. The dataset was divided as follows, at random: 46 examples were used for training, and another 5 were used for testing.

#### 3.1.2. Data Augmentation

**Figure 2:**
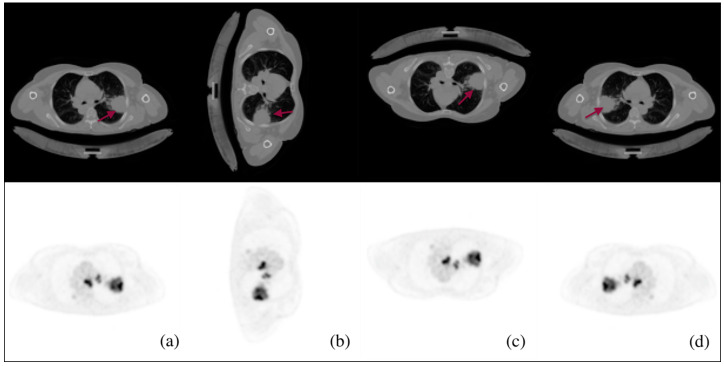
Some examples of the augmentation process of CT and PET images for STS: (**a**) the main CT-PET, (**b**) rotating the CT-PET by 90 degrees clockwise, (**c**) flipping the CT-PET upside down, and (**d**) left-mirroring the CT-PET. Red arrows indicate the tumour region.

The CT-PET images were augmented throughout this phase to prevent overfitting, which helps in enhancing the performance of the model. In addition, the implementation of augmentation techniques, such as random rotations, flips, and cuts, can enhance the model’s ability to maintain invariance towards variations in feature position and orientation within the image. This feature proves to be particularly advantageous when working with real-world images that may exhibit variations in object orientation or spatial arrangement. Images are augmented in three ways, as shown in Figure 2: rotating the CT-PET by 90 degrees clockwise (2b), flipping the CT-PET upside down (2c), and left-mirroring the CT-PET (2d).

#### 3.1.3. Image Post-Processing

The suggested framework’s ultimate stage used a morphological change and a basic thresholding technique. To lessen the impact of noise, a morphological gradient accounted for the structure of the input picture. Its effect is analogous to the difference between expanding and contracting an image.

While Equation (1) defines dilation [21] as the process of removing pixels (noises) from object boundaries, Equation (2) describes erosion [22] as the process of adding pixels (negative noises) to object boundaries.

(1) $$A⨁B=\cup_{b\in B}A_{b}$$

(2) $$A!B=\left\{z\in E|B\subseteq A\right\}$$

where A is a set of pixels, and *B* is a structuring element. The thresholding technique is defined as Equation (3).

(3) $$f\left(x,y\right)=\left\{\begin{array}{c} 1\;\;\;\;\;\;if\;x>t\\ 0\;\;\;\;\;if\;x<t \end{array}\right.$$

where t is the threshold value employed to determine if the pixel concerned is part of the desired ground truth or the background. These methods are then used on the expected ground truth to soften the edges and remove false positive values. After being processed, the images of tumour tissue are sharp and in sharp focus.

**Figure 3:**
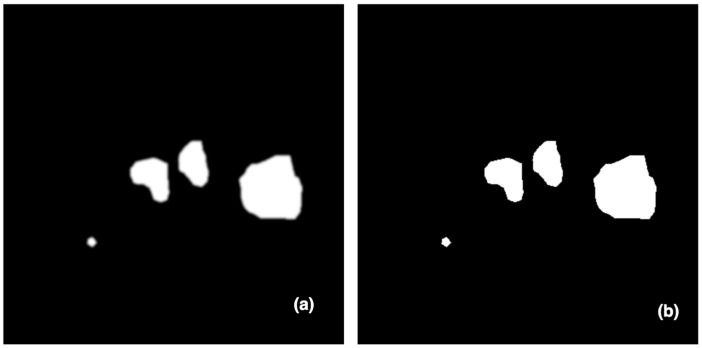
An example of the post-processing effect: (**a**) predicted mask and (**b**) image after mask post-processing.

Figure 3 depicts the last stage in processing predicted masks, in which tiny false positive values and blobs at the borders are removed.

#### 3.1.4. Multimodal Feature Fusion

A feature fusion strategy is deployed in medical imaging to generate a higher quality final image. Feature extraction, classification, and decision making are the three main pillars of any supervised learning-based method. To broaden the types of features collected and better understand their relationships, the early and late sequences of feature fusion are employed in the encoder portion of the core U-Net design. Features from different imaging modalities, like PET and CT, are fused serially to better characterize lung tumours.

#### 3.1.5. Early Fusion

**Figure 4:**
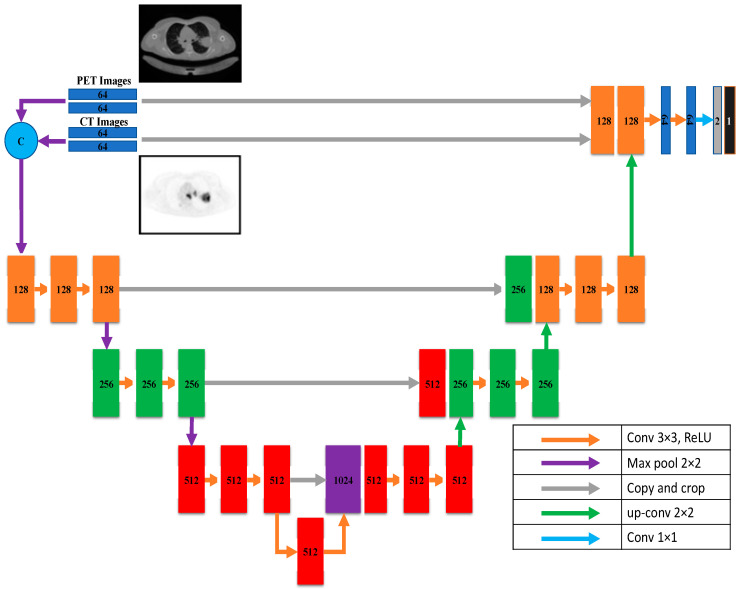
Early fusion architecture.

In early fusion, each medical image scan (CT and PET) has a single input path which contains two CNN layers with 64 units and a Relu activation function. Then, these two paths are concatenated into a single path, which is processed through a unique path in the down-sampling U-Net path. This path contains three groups of CNN architecture; each group has three CNN layers with 128, 256, and 512 units, followed by a max pooling layer. All CNNs activation functions are Relu functions. Figure 4 shows the early fusion architecture.

#### 3.1.6. Late Fusion

**Figure 5:**
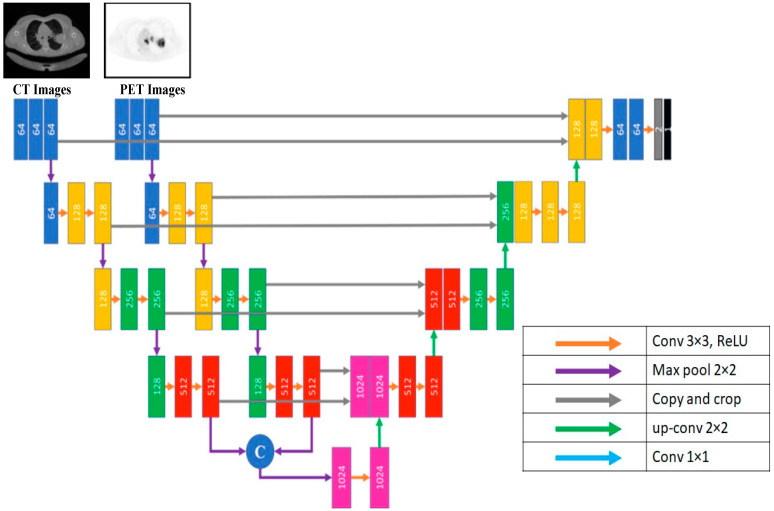
Late fusion architecture.

In contrast to most architectures like U-Net, the encoding path is divided into N streams that serve as input for each imaging modality. Each modality learns a unique feature set using images from the other. The two modalities’ feature maps are combined at each network’s high-level feature layer. This process solves the problem of the early fusion strategy. These feature sets are combined into one feature set and then subjected to the last phase of a multimodal classifier’s training. The U-Net down-sampling path contains four groups of CNN layers. Each group contains three sequential CNN layers with 64, 128, 256, and 512 units, respectively, followed by a max pooling layer. All CNNs have a Relu activation function. At this point, the two paths are concatenated to generate the input of the U-Net up-sampling path. Figure 5 shows the late fusion architecture.

#### 3.1.7. Dense Fusion

**Figure 6:**
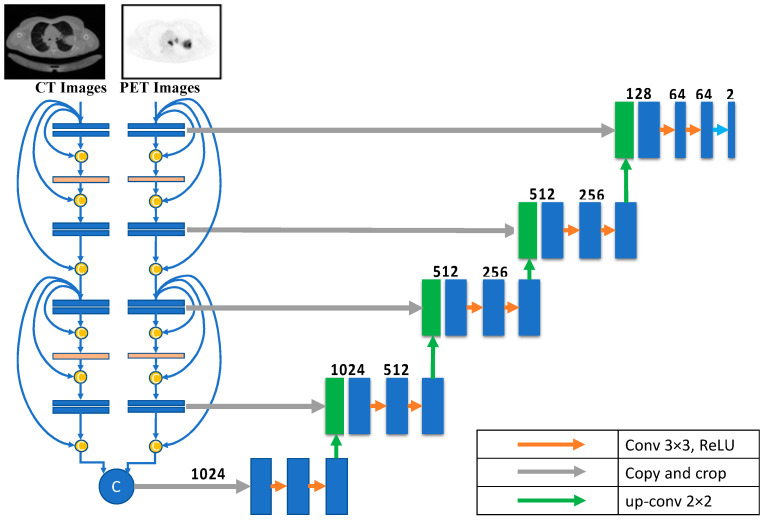
Dence fusion architecture.

For lung cancer segmentation, the dense-fusion-based U-Net provides two down-sampling routes, one for CT and one for PET images. Eight CNN deep learning building blocks are used along each possible route. All layers preceding the current layer are inputs to the current CNN layer. A max-pooling layer follows each pair of consecutive CNN layers. The dimensions of the CNN layer are (in order) 64, 128, 256, and 512. The Relu activation function is standard in all CNNs. The input to the U-Net up-sampling path is generated by concatenating the outputs of the paths following the design described in each path. The dense fusion architecture is shown in Figure 6.

#### 3.1.8. Hyper-Dense Fusion

**Figure 7:**
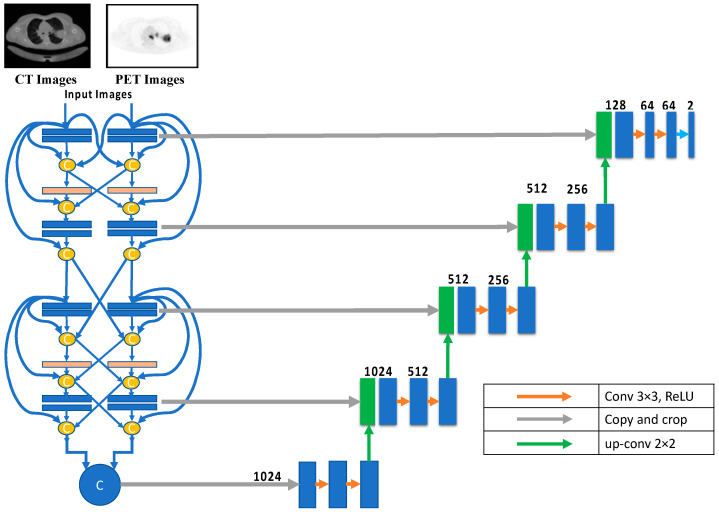
Hyper-dense fusion architecture.

Deep learning is essential when an application requires a deep layer to function effectively and efficiently. Reducing the overfitting impact is one of several benefits of using dense architecture for multimodality U-Net medical image segmentation. The layers in the same input path provide inputs to all net layers for dense design, which is necessary for U-Nets with multiple input paths. Each layer feeds its immediate successor and those in adjacent input channels in hyper-dense fusion. As the network learns the intricate connections between the modalities at each level of abstraction, the hyper-dense connectivity produces a more robust feature representation than early/late fusion in a multimodal situation. The hyper-dense fusion layout is depicted in Figure 7.

### 3.2. Loss Functions

In the proposed method, we thoroughly investigated and compared the models using a variety of loss functions. Segmenting an image is essentially a pixel-level classification problem. Each pixel in an image contributes to the overall image, and specific clusters of pixels define particular aspects. Semantic image segmentation is a technique that divides these pixels into their respective components. While designing intricate, deep learning architectures for image segmentation, choosing the loss/objective function is crucial. Loss functions can be broken down into several types based on distribution, region, boundary, and compound. The proposed analysis uses three distinct loss functions, i.e., binary cross-entropy, Dice, and focal. The representation as the network discovers the many interconnections between modalities at every level of abstraction, rather than the binary early/late fusion approach.

#### 3.2.1. Binary Cross-Entropy

Cross-entropy [23] is used to quantify the dissimilarity between two probability distributions for a specific random variable or sequence of events. Segmentation, being a categorization at the pixel level, is extensively employed for this purpose, and it achieves good results. The formula for cross-entropy is given in Equation (4).

(4) $$L_{BCE\left(y,\hat{y}\right)}=-\left[y\log\left(\hat{y}\right)+\left(1-y\right)\log\left(1-\hat{y}\right)\right]$$

where $\hat{y}$ is the predicted value by the trained model.

#### 3.2.2. Focal Loss

Focal loss (FL) [24] can be understood as a form of binary cross-entropy. It reduces the importance of simple instances, letting the model devote more time and energy to mastering complex ones. It is effective when there is a significant imbalance between the classes. The formula for focal loss is given in Equation (5)

(5) $$FL\left(pt\right)=-\alpha t{\left(1-pt\right)}^{\gamma }\log\left(pt\right)$$

where ${\left(1-p_{t}\right)}^{\gamma }$ is the modulating factor. Here, γ > 0, and when γ = 1, the focal loss will be the same as the cross-entropy loss. Similarly, γ can be set by the inverse class frequency or used as a hyperparameter with a typical range of [0, 1].

#### 3.2.3. Dice Loss

In computer vision, the Dice coefficient is a metric utilized extensively to calculate the degree of similarity between two images. In the latter half of 2016, it was also adapted as a Dice loss function [25]. The formula for Dice loss is given in Equation (6).

(6) $$FDL\left(y,\;\hat{p}\right)=1-\frac{\left(2y\hat{p}+1\right)}{\left(y+\hat{p}+1\right)}$$

In this case, 1 is added to both the numerator and denominator to prevent the function from being undefined in exceptional cases like $y=\hat{p}=0$.

## 4. Hyper-Dense VGG16 U-Net Segmentation Proposed Model

In various computer vision problems, shortcut connections between layers have become increasingly popular since the emergence of residual learning [26]. Unlike in conventional networks, these links back-propagate gradients immediately, which helps prevent gradient-vanishing issues and allows for more complex architectures. The idea of shortcut connections was expanded upon by DenseNet [27], which specified that each layer’s inputs should correspond to the outputs of all layers that came before them. Densely connected convolutional neural networks (CNNs) are built using the feed-forward principle, which entails adding direct connections from any layer to all succeeding layers. Deep networks are more accessible and more accurate to train because of this connectivity. This section proposes independently expanding U-Net to support DenseNet connections within the same multiple N streams of PET and CT modalities. Higher level layers of the proposed extension will also use the late fusion strategy.

The inspiration for this comes from three separate observations. First, all architectural feature maps are connected by short paths, enabling implicit deep supervision. Second, the network’s information and gradients are better able to flow because of the direct connections between all layers. Finally, the regularizing effect of dense connections makes it less likely that training data will be too small for a given task.

Using dense and hyper-dense connections has been demonstrated to have many benefits when segmenting medical images. More information can be gleaned from medical images when the VGG architecture is used for feature extractions. We propose a multimodality U-Net medical image segmentation model using hyper-dense connections and the VGG16 model.

Our primary objective was to refine an existing deep-learning model for lung cancer segmentation. To do this, we modified the U-Net design and used it as the starting point. The encoder and the decoder are both CNNs, making up the basic U-Net architecture. The encoder extracts features by first performing convolutional operations and then down-sampling. The usual convolutional processes follow the up-sampling and concatenation layer of the decoder branch. Connecting feature maps from the encoder network is made possible via a skip link that connects the same-level layers of the decoder and encoder, with the up-sampled feature map conveying coarse global context information. This helps with recovering local characteristics after down-sampling.

**Figure 8:**
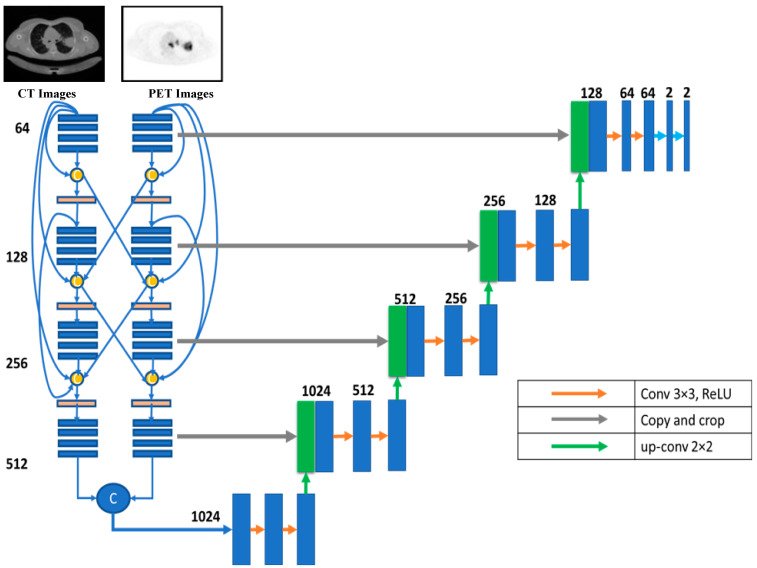
The proposed hyper-dense VGG16 U-Net model architecture.

According to this model, U-Net takes data via two distinct input paths, one for each image type. The architecture of both paths is VGG16, with dense and hyper-dense connections between them. This architecture was proposed so that image classification and segmentation tasks may take advantage of VGG, dense, and ultra-dense networks. The suggested VGG16 U-Net model’s components are given in Figure 8.

Figure 8 depicts the proposed hyper-dense VGG16 U-Net model, built upon the U-Net. Both CT and PET images can be fed into the model. The segmented image of lung cancer is the product of the model. In the suggested approach, input images for both CT and PET were 128 × 128. Each image input type has its dedicated input path, each with 16 CNNs (the number of CNNs in VGG16). Each dataset was processed through CNNs of varying sizes (64, 128, 256, and 512). Figure 6 shows that both input paths are incredibly well connected, and there are also many connections between the two. All convolutional neural networks used ReLU activation. The decoder’s structure comprises four groups of convolutional neural networks (CNNs) of varying sizes (1024, 512, 256, and 128).

## 5. Experiments

The efficiency of the proposed U-Net models for segmenting lung tumours was measured across various performance criteria. The STS dataset was used for both training and testing the models. Experiments compared the newly developed models to benchmarked models widely utilized on the same dataset and other datasets.

### 5.1. Experimental Setup

All experiments were run on servers in the Google Colaboratory environment, and the recommended models for segmenting lung tumours were built using a TensorFlow and Keras backend with an NVIDIA Tesla P100 -PCIE GPU and 32.0 GB RAM. For the training phase, we employed the Adam optimizer with the following settings: learning rate = 0.0001, 1 = 0.9, 2 = 0.999, and epsilon = 1 × 10^−8^. One hundred epochs of training were used. The intensity levels of the image’s pixels were normalized to remove any potential for ambiguity. The dataset was arbitrarily divided into 70% for training, 20% for validation, and 10% for testing.

### 5.2. Dataset Description

The proposed models are trained on data from a study of soft-tissue sarcomas (STSs) [28]. STS includes many types of scans, such as CT, PET, and MRI, but in our research only the CT and PET scans were used. In this dataset, a cohort of 51 patients with histologically proven soft-tissue sarcomas (STSs) of the extremities was retrospectively evaluated. It included 27 females and 24 males, with ages ranging from 16 to 83 years old, and with various cancer degrees: low, intermediate, and high. The PET slice volumes had a thickness of 3.27 mm and a median in-plane resolution of 5.47 mm × 5.47 mm (range: 3.91–5.47 mm). All images used in our tests were downsized to 128 pixels on the longest dimension. For our experiments, we excluded slices without tumour pixels in the ground truth. In total, over 3000 pairs of PET-CT image slices (3063 STS) were included.

### 5.3. Performance Metrics

The efficiency of the suggested approach was assessed using the most commonly employed metric for evaluating segmentation tasks [29]: the Dice score (Dice), the most crucial segmentation performance measure. It is defined by Equation (7).

(7) $$Dice=\frac{\left(2\;*\;Tp\right)}{\left(2\;*\;Tp+Fp+Fn\right)}$$

In addition, measures of accuracy, sensitivity, and specificity were used. Equations (8) to (10) also provide definitions for them.

(8) $$Accuracy=\frac{Tp+Tn}{Tp+Tn+Fn+Fp}$$

(9) $$Sensitivity=\frac{Tp}{Tp+Fn}$$

(10) $$Specificity=\frac{Tn}{Tn+Fp}$$

where the four primary blocks for computing these metrics were defined as true positive (Tp), true negative (Tn), false positive (Fp), and false negative (Fn) values.

## 6. Results and Discussion

The effectiveness of the proposed models is discussed in this section. In this section, we report the results of our model assessments, broken down into four categories: loss function comparisons, same-dataset comparisons, cross-dataset comparisons, and cross-model comparisons. Dice, IoU, accuracy, spectral sensitivity, and area under the curve (AUC) were utilized as performance measures.

### 6.1. Loss-Function-Based Comparison

Adjustments to the loss functions form the basis for a new comparative evaluation of the models. Focal loss functions, Dice, and binary cross-entropy are employed in our research. These operations are among the most well-known and often used in deep learning for image segmentation. The outcomes are displayed in Table 2, Table 3 and Table 4 for binary, Dice, and focal loss functions.

**Figure 9:**
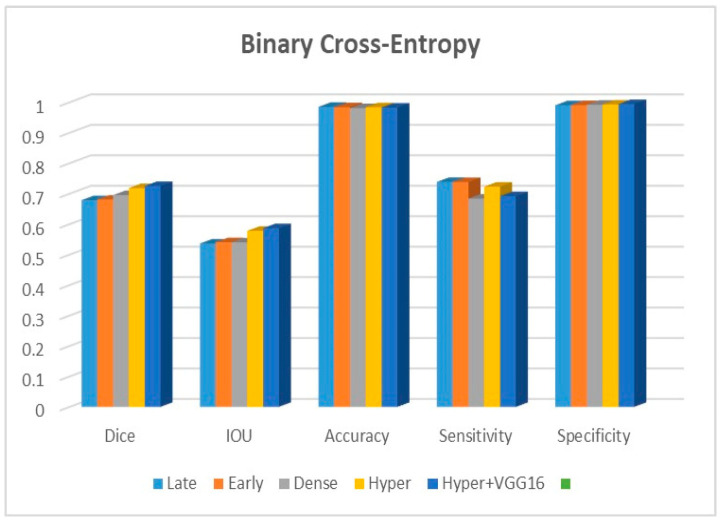
Binary cross-entropy function.

**Figure 10:**
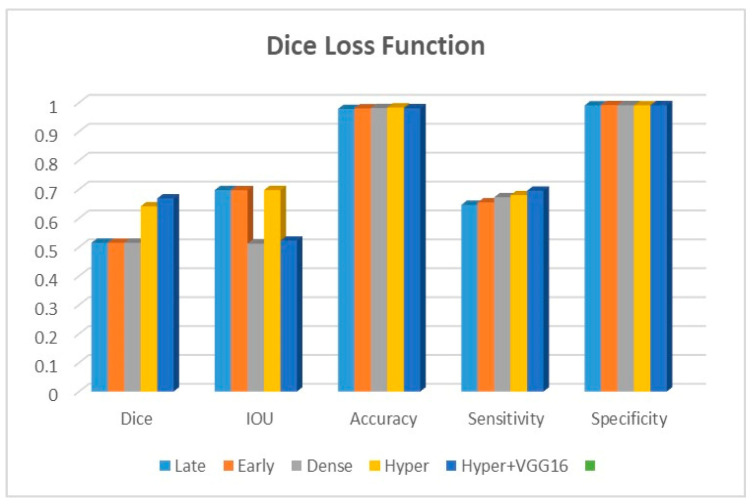
Dice loss function.

**Figure 11:**
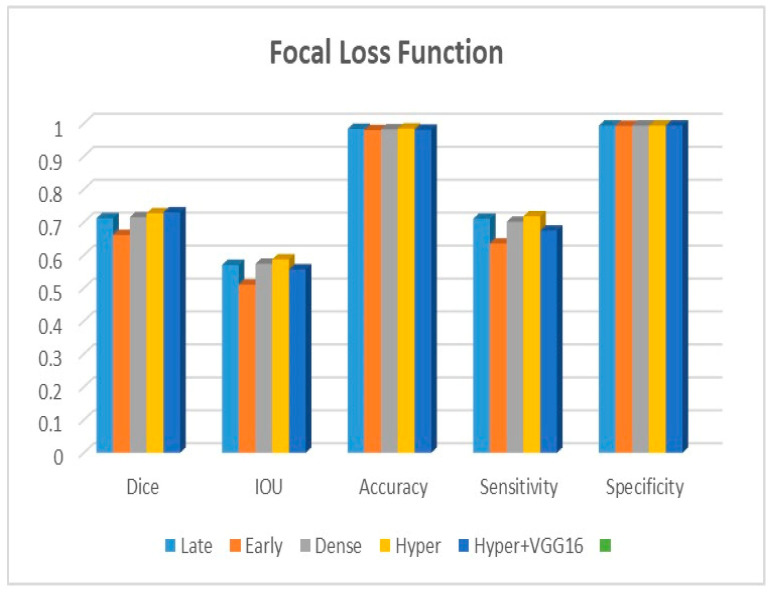
Focal loss function.

**Figure 12:**
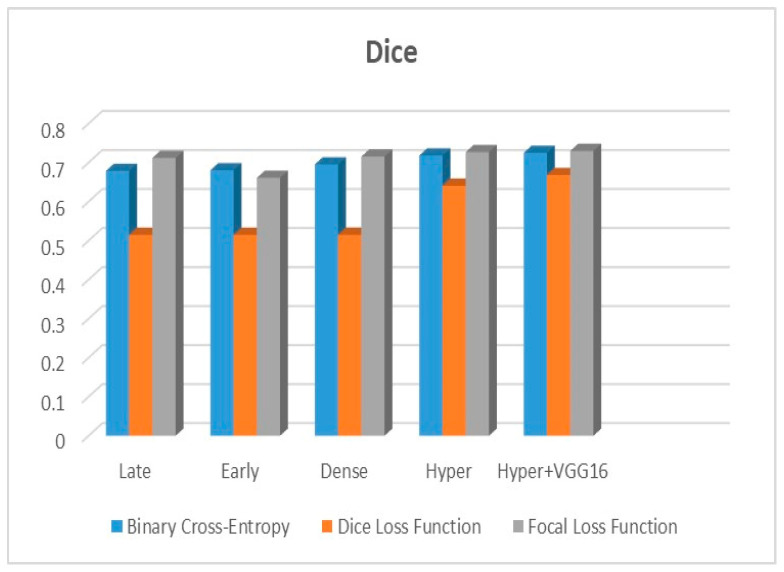
Dice metric.

**Figure 13:**
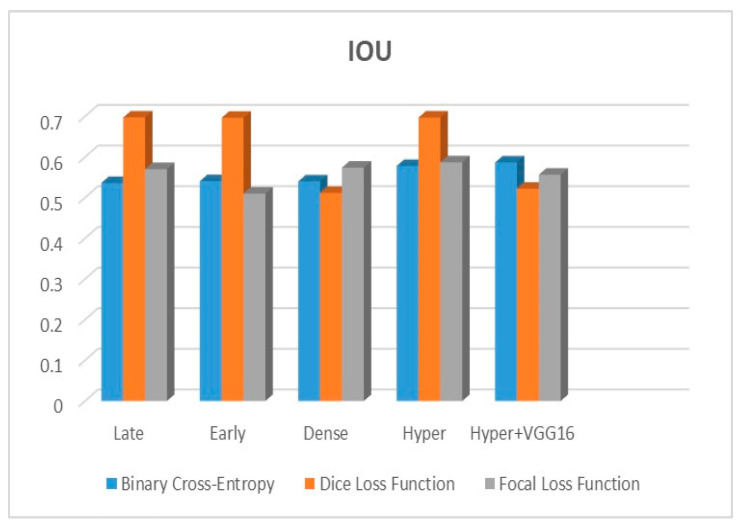
IOU metric.

**Figure 14:**
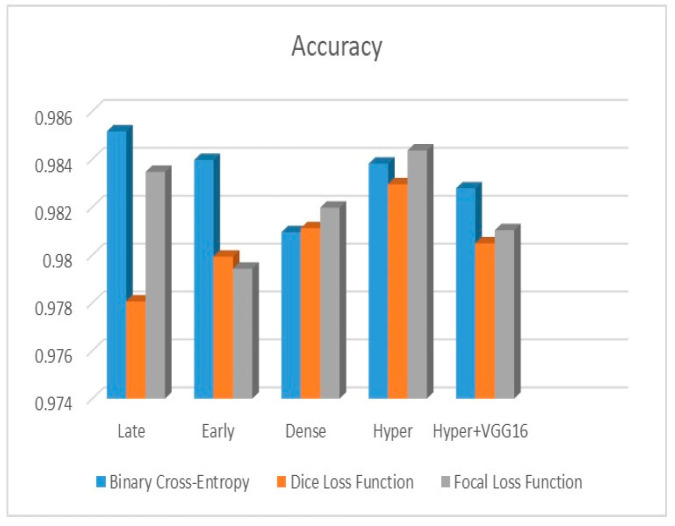
Accuracy metric.

**Figure 15:**
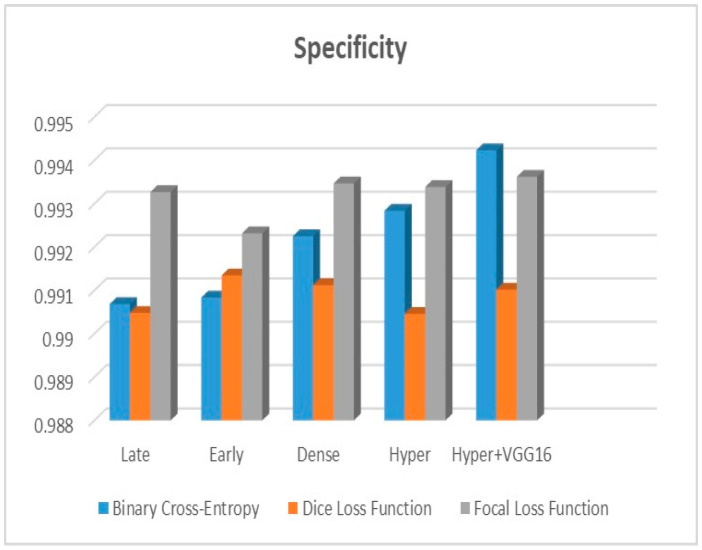
Specificity metric.

**Figure 16:**
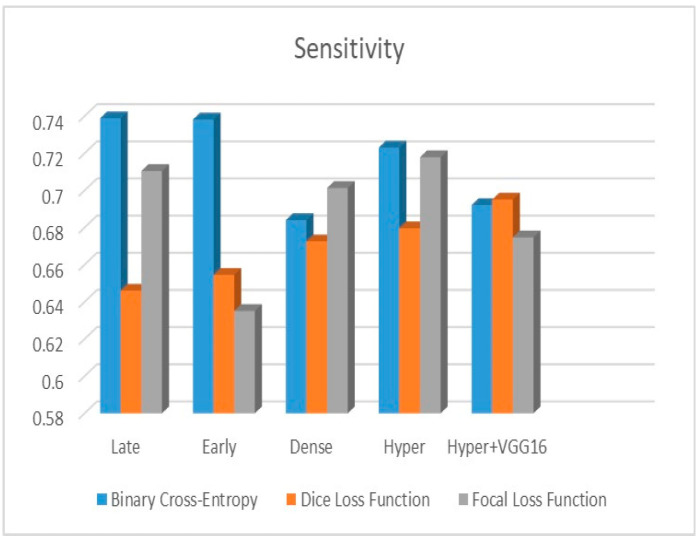
Sensitivity metric.

Figure 9, Figure 10 and Figure 11 depict the findings using various loss functions, like cross-entropy, focal loss, and Dice loss. In contrast, the performance measures for the proposed models using the metrics Dice, IoU, accuracy, sensitivity, and specificity are given in Figure 12, Figure 13, Figure 14, Figure 15 and Figure 16, respectively.

**Figure 17:**
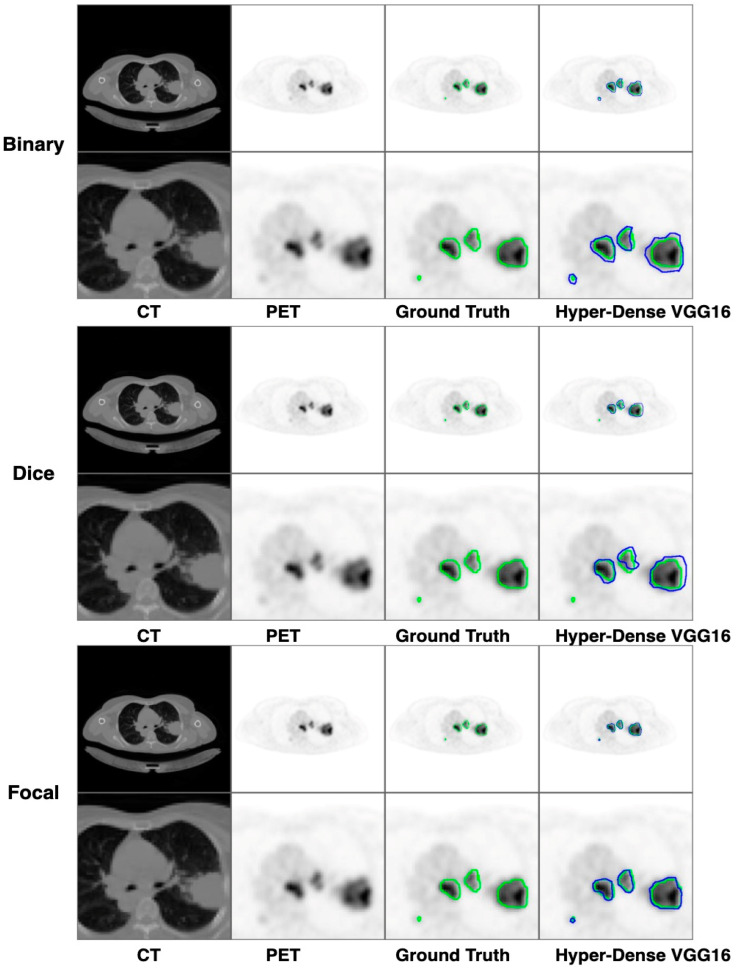
The comparison of lung tumour segmentation results, along with the segmentation outcomes for corresponding enlarged tumour regions, using our proposed hyper-dense VGG16 model with various loss functions (“Binary”, “Dice”, and “Focal”). The green contours outline the “Ground Truth” segmentation, and the blue contours outline results from the proposed model.

The suggested hyper-dense VGG16 model outperforms the other models in Dice for all types of loss functions, as seen in Table 2, Table 3 and Table 4.The focal loss function is the only option if one wants the best Dice performance possible. Figure 9, Figure 10, Figure 11, Figure 12, Figure 13, Figure 14, Figure 15 and Figure 16 offer graphical representations of the evaluation outcomes. The five presented models are compared in Figure 9, Figure 10, Figure 11 and Figure 12 regarding the binary cross-entropy, Dice, and focal loss functions used as performance indicators. Figure 9 shows that the suggested hyper VGG16 model outperforms the others in terms of Dice accuracy (improved by 7%), IOU accuracy (improved by 9%), and specificity accuracy (improved by 0.4%). However, the late fusion model’s accuracy and sensitivity are unparalleled. The results of the Dice loss function are shown in Figure 10, and it is evident that the suggested model outperforms the previously introduced models in terms of Dice, specificity, and sensitivity. Finally, the proposed model outperforms the other established models regarding the focal loss function performance, achieving 73% for Dice. Figure 10, Figure 11, Figure 12 and Figure 13 present visual representations of the performance above metrics about the loss function employed. Figure 13 demonstrates that the most outstanding value for the focal loss function is found with the Dice metric. The segmentation results of the proposed model for various loss functions are displayed in Figure 17. In Figure 17, the lung tumour segmentation results generated by hyper-dense VGG16 are compared to the ground truth, employing various loss functions such as binary, Dice, and focal. The observations from Figure 17 indicate that the focal loss function yields the most accurate predictions, capturing even the segmentation of small tumour portions and producing a predicted segmentation mask that closely aligns with the ground truth segmentation. Conversely, when utilizing the binary cross-entropy loss function, the segmentation results tend to be slightly larger. The Dice loss function, however, provides the least accurate predictions, as it fails to segment small tumour portions and produces a larger overall segmentation compared to the ground truth. 

### 6.2. Same Dataset Models in the State of the Art

**Figure 18:**
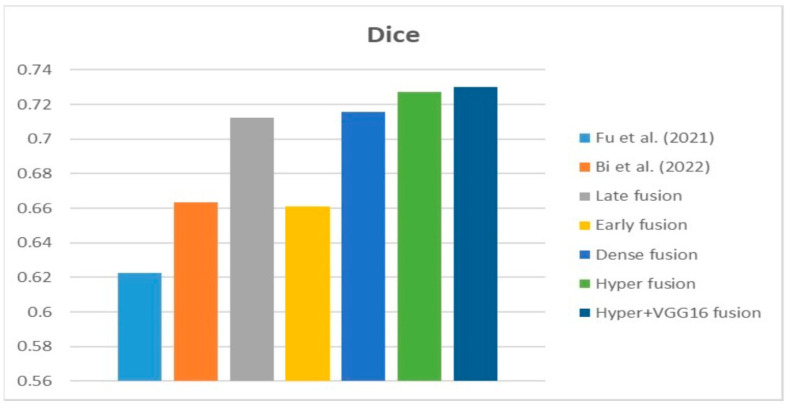
Dice [13,20].

**Figure 19:**
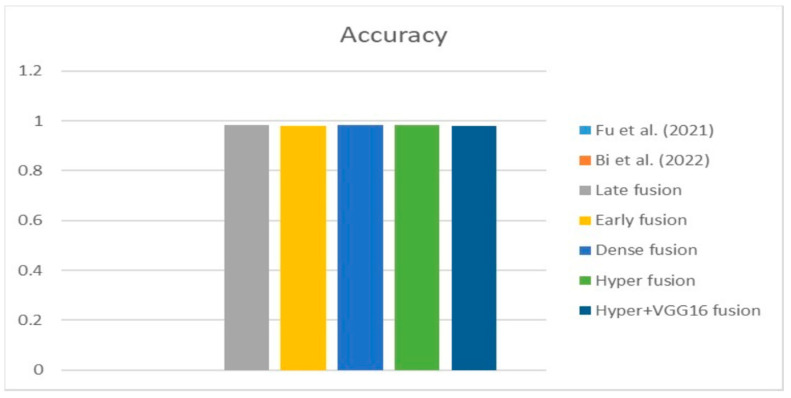
Accuracy [13,20].

**Figure 20:**
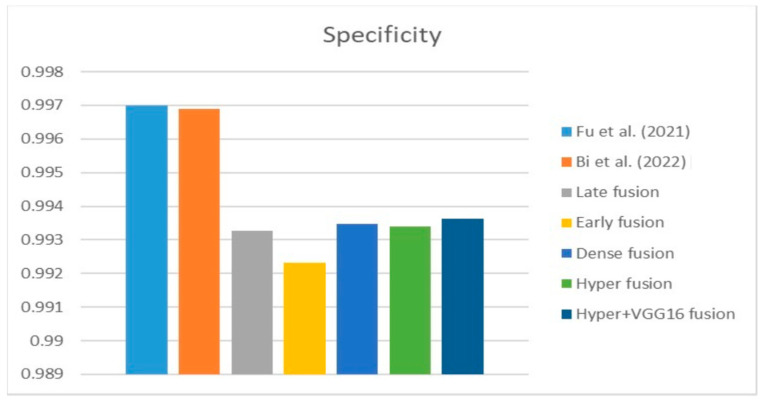
Specificity [13,20].

**Figure 21:**
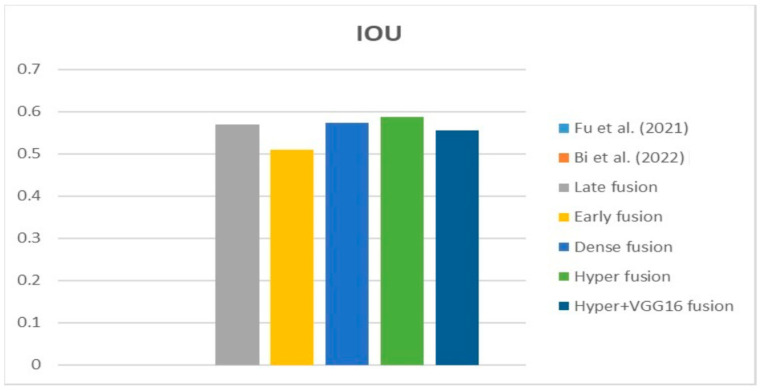
IOU [13,20].

**Figure 22:**
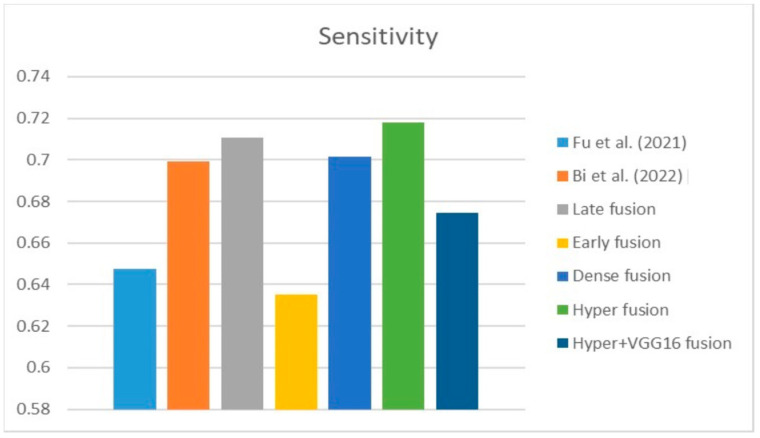
Sensitivity [13,20].

Table 5 compares the Dice, IoU, accuracy, specify, and sensitivity scores of the proposed model to those of the benchmarked research conducted on the same STS dataset. As shown in Table 5, the dense fusion model outperforms the other models using the same dataset. Figure 18, Figure 19, Figure 20, Figure 21 and Figure 22 shown visual representations of the performance metrics which were used in performance evaluation and the comparison of the proposed models.

According to the data in the table and the graphs above, the suggested hyper-dense VGG16 model outperforms state-of-the-art models trained on the same dataset by a margin of Dice improvement equal to 17%. The proposed architecture for feature extraction between the two types of inputs and applying the VGG16 architecture of image feature extraction led to this enhancement.

### 6.3. Different Datasets in the State of the Art

**Figure 23:**
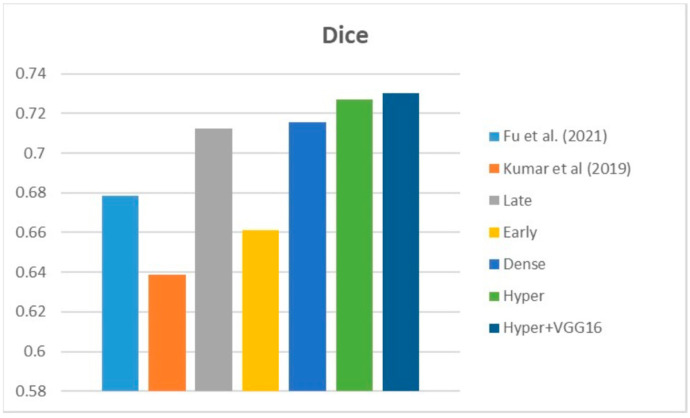
Dice [13,16].

**Figure 24:**
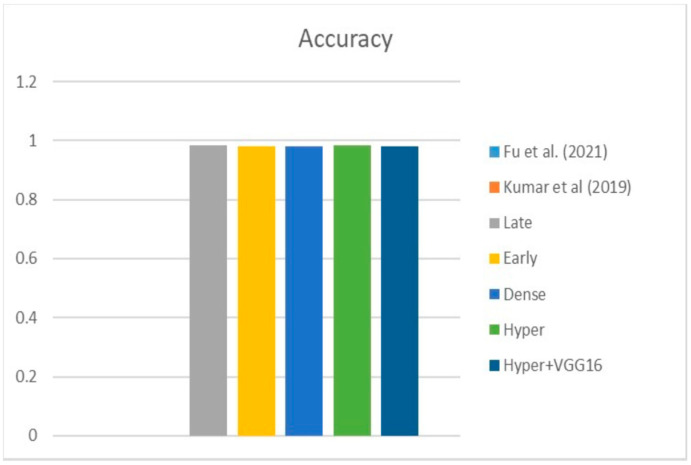
Accuracy [13,16].

**Figure 25:**
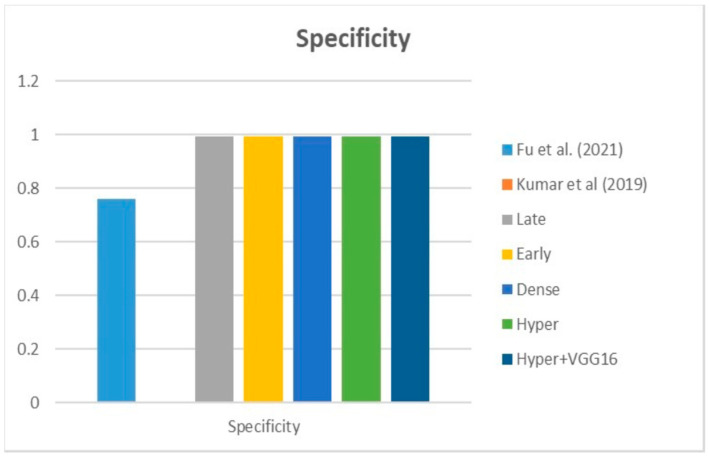
Specificity [13,16].

**Figure 26:**
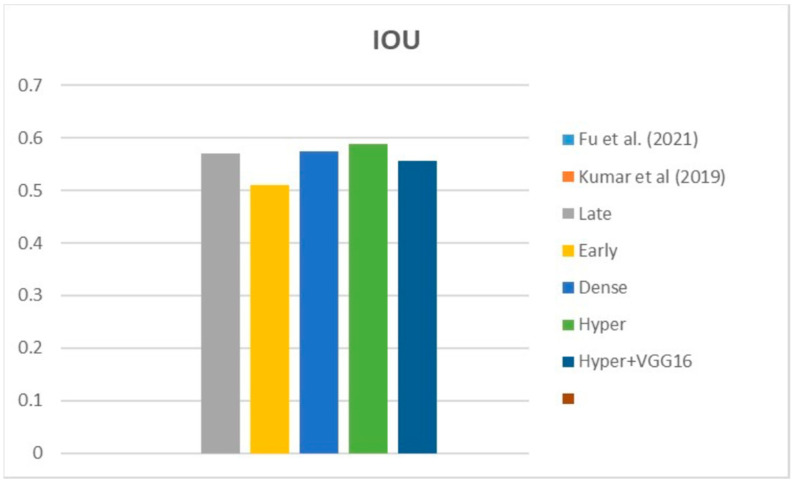
IOU [13,16].

**Figure 27:**
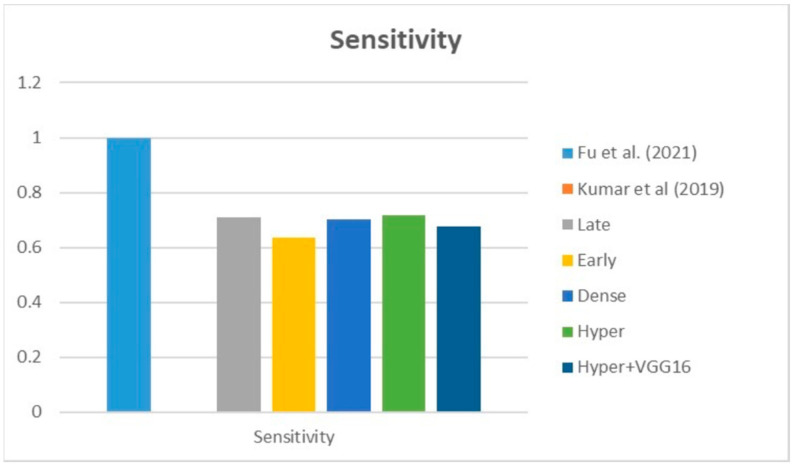
Sensitivity [13,16].

Table 6 compares the Dice, IOU, accuracy, specificity, and sensitivity metrics of the proposed model to those of benchmarked studies that employed different datasets, demonstrating the model’s superior performance. Table 6 show that the dense fusion model has a higher Dice value than the other models. Figure 23, Figure 24, Figure 25, Figure 26 and Figure 27 show visual representations of the performance metrics used in performance evaluation and the comparison of the proposed models.

Based on the data in the table, we infer that the suggested hyper-dense VGG16 model outperforms the other state-of-the-art lung cancer segmentation models trained on a separate dataset by a margin of Dice improvement equal to 14%. The proposed architecture for feature extraction between the two types of inputs and applying the VGG16 architecture of image feature extraction led to this enhancement.

## 7. Conclusions and Future Scope

Despite significant tumour detection and treatment developments, lung cancer remains a leading cause of mortality globally. This study offers a deep learning model based on hyper-dense VGG16 deep technologies to segment lung cancer using U-Net as a backbone structure with multimodal input images. A comprehensive evaluation of the proposed model was included: introducing four models to compare with the proposed one, comparing the proposed model with others presented in the state of the art, and comparing the proposed model with the others using the same and different datasets. The hyper-dense VGG16 model performed the best out of all the analyses performed on this dataset, receiving a Dice score of 73%. The hyper-dense VGG16 model achieved better performance because the extraction of features in each layer is not only a function of the previous layer input, but a function of all previous inputs in the same input branch, and it has inputs from the other input branches which make the feature extraction deeper and more accurate. However, further research based on the outcomes of this study can go in a few different directions, such as employing a combination of CT, PET, and MRI as input to the segmentation model to verify the suggested model’s generalizability and introducing the performance study of the proposed model with respect to a combination of two types of loss functions such as binary and Dice loss functions. Furthermore, the model’s performance can be tested by experimenting with various hyperparameter values of the offered methodologies.

## Figures and Tables

**Table 1:** Literature summary.

Author	CT-Only Extractor	PET-Only Extractor	Feature Fusion	Dataset Description
Wang et al. [10]	3D CNN	3D CNN	3D CNN	Private clinic dataset comprising 290 pairs of CT and PET.
Park et al. [11]	Global U-Net	Global U-Net	Regional U-Net	Private data of 887 individuals with lung cancer.
Xiang et al. [12]	Dual-stream encoder	Dual-stream encoder	Decoder branch	126 PET-CT scans containing NSCLC
Fu et al. [13]	Encoder-decoder backbone CNN	Multimodal spatial attention module (MSAM).	CNN architecture containing skip connections.	Two clinical PET-CT datasets of NSCLC and STS.
Zhong et al. [14]	3D U-Net	3D U-Net	Graph-cut-based co-segmentation model	PET-CT scans from lung cancer patients.
Hwang et al. [15]	Shared co- encoder	Shared co-encoder	Shared co-encoder	F-18-FDG PET/CT scans from a private hospital.
Kumar et al. [16]	Encoder using multiscale output	An encoder using multiscale output	Decoder using multiscale multimodal input	Biopsy-proven NSCLC FDG PET-CT scans.
Jemaa et al. [17]	-	-	2D U-Net and selected VNet	Patients with non-Hodgkin’s lymphoma and NSCLC, which includes 3664 FDG-PET/CT images from head to toe.
Zhao et al. [18]	VNet	VNet	Voxel-wise addition, along with VNet	Private clinical dataset having 3D PET/CT images.
Zhong et al. [19]	Encoder using multiscale output	An encoder using multiscale output	Decoder using multiscale multimodal input	NSCLC patients who received stereotactic radiation treatment.
Bi et al. [20]	CNN-TN Encoder	CNN-TN Encoder	TN-CNN decoder	Non-small-cell lung cancer (NSCLC) and one soft-tissue sarcoma (STS) dataset.

**Table 2:** Binary cross-entropy.

	Dice	IOU	ACC	Sen	Spec
Late	0.67882	0.53651	0.98516	0.73885	0.99068
Early	0.68066	0.54075	0.98397	0.73816	0.99083
Dense	0.69569	0.54016	0.98095	0.68401	0.99225
Hyper	0.71851	0.57818	0.98381	0.72302	0.99284
Hyper + VGG16	0.72532	0.58687	0.98278	0.69209	0.99423

**Table 3:** Dice loss function.

	Dice	IOU	ACC	Sen	Spec
Late	0.51479	0.69734	0.97806	0.64604	0.99048
Early	0.51465	0.69671	0.97993	0.65452	0.99135
Dense	0.51485	0.51191	0.98112	0.67253	0.99112
Hyper	0.64081	0.69725	0.98295	0.67958	0.99046
Hyper + VGG16	0.66828	0.52222	0.98048	0.69506	0.99102

**Table 4:** Focal loss function.

	Dice	IOU	ACC	Sen	Spec
Late	0.71217	0.5704	0.98347	0.71046	0.99327
Early	0.66112	0.51011	0.97943	0.6351	0.99232
Dense	0.71554	0.57403	0.98198	0.70131	0.99347
Hyper	0.72713	0.58717	0.98436	0.71786	0.99339
Hyper + VGG16	0.73011	0.55664	0.98103	0.67472	0.99362

**Table 5:** Comparison of the proposed and benchmarked models on the STS dataset.

	Dice	IOU	ACC	Sen	Spec
Fu et al. [13]	62.26	-	-	64.74	99.7
Bi et al. [20]	66.36	-	-	69.93	99.69
Late	0.712171	0.5704	0.98347	0.71046	0.99327
Early	0.661116	0.51011	0.97943	0.6351	0.99232
Dense	0.715539	0.57403	0.98198	0.70131	0.99347
Hyper	0.72713	0.58717	0.98436	0.71786	0.99339
Hyper + VGG16	0.73.0109	0.55664	0.98103	0.67472	0.99362

**Table 6:** Comparison of the proposed and benchmarked models on different datasets.

	Dice	IOU	ACC	Sen	Spec
Fu et al. [13]	67.83	-	-	99.9	76.16
Kumar et al. [16]	63.85	-	-	-	-
Late	0.712171	0.570397	0.983471	0.710462	0.993269
Early	0.661116	0.510114	0.979428	0.635095	0.992316
Dense	0.715539	0.57403	0.981976	0.701314	0.993468
Hyper	0.72713	0.587171	0.984362	0.717861	0.993387
Hyper + VGG16	0.730109	0.556635	0.981034	0.674717	0.99362

## Data Availability

The dataset used for this study is publicly available and contained within the article.
